# Large‐scale molecular barcoding of prey DNA reveals predictors of intrapopulation feeding diversity in a marine predator

**DOI:** 10.1002/ece3.6638

**Published:** 2020-08-26

**Authors:** Madelyn R. Voelker, Dietmar Schwarz, Austen Thomas, Benjamin W. Nelson, Alejandro Acevedo‐Gutiérrez

**Affiliations:** ^1^ Biology Department Western Washington University Bellingham WA USA; ^2^ Smith‐Root Vancouver WA USA; ^3^ Institute for the Oceans and Fisheries University of British Columbia Vancouver BC Canada; ^4^ Present address: Ocean Research College Academy Everett WA USA

**Keywords:** intrapopulation feeding diversity, pinniped, predator–prey interactions, proportional similarity index

## Abstract

Predator–prey interactions are critical in understanding how communities function. However, we need to describe intraspecific variation in diet to accurately depict those interactions. Harbor seals (*Phoca vitulina*) are an abundant marine predator that prey on species of conservation concern. We estimated intrapopulation feeding diversity (variation in feeding habits between individuals of the same species) of harbor seals in the Salish Sea. Estimates of feeding diversity were examined relative to sex, month, and location using a novel approach that combined molecular techniques, repeated cross‐sectional sampling of scat, and a specialization metric (within‐individual consistency in diet measured by the Proportional Similarity Index ($PS_{i}$)). Based on 1,083 scat samples collected from five haul‐out sites during four nonsequential years, we quantified diet using metabarcoding techniques and determined the sex of the scat depositor using a molecular assay. Results suggest that intrapopulation feeding diversity was present. Specialization was high over short periods (24–48 hr, $PS_{i}$ = 0.392, 95% CI = 0.013, R = 100,000) and variable in time and space. Females showed more specialization than males, particularly during summer and fall. Additionally, demersal and benthic prey species were correlated with more specialized diets. The latter finding suggests that this type of prey likely requires specific foraging strategies and that there are trade‐offs between pelagic and benthic foraging styles for harbor seals. This differential feeding on prey species, as well as between sexes of harbor seals, indicates that predator–prey interactions in harbor seals are complex and that each sex may have a different impact on species of conservation concern. As such, describing intrapopulation feeding diversity may unravel hitherto unknown complex predator–prey interactions in the community.

## Graphical abstract

We estimated intrapopulation feeding diversity of harbor seals in the Salish Sea relative to sex, time, and location with a novel approach that combined molecular techniques, repeated cross‐sectional sampling of scat, and a specialization metric (within‐individual consistency in diet). Differential feeding on prey species was revealed, as well as differences between sexes of harbor seals. This indicates that predator–prey interactions in harbor seals are complex and that each sex may have a different impact on species of conservation concern. As such, describing intraspecific variation in diet may unravel hitherto unknown complex predator–prey interactions in the community.

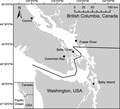

## 1 INTRODUCTION

Predator–prey relations are an integral force in ecosystem functions and are often key to understanding how communities interact. Predators occupy a wide spectrum of foraging strategies, ranging from generalists to specialists. Individuals using a smaller subset of resources than the population as a whole are defined as individual specialists (Van Valen, 1965) whereas individuals consuming a wider range of resources than used on average by the population are defined as generalists (Hanski, Hansson, & Henttonen, 1991). In some cases, preying on one species may preclude consumption of a different species as there may be trade‐offs in skills required to utilize different resources (Arthur et al., 2016; Wilson & Yoshimura, 1994). Interindividual differences in resource use that are transient and the result of short‐term choices in habitat or hunting strategies are best described as intrapopulation feeding diversity while permanent differences between individuals based on sex, size, or personality are better described as individual specialization (Van Valen, 1965). Both types of interindividual differences in resource use need to be examined to understand predator–prey interactions.

The level of individual specialization and/or intrapopulation feeding diversity can affect food web dynamics, responses to changes in prey availability, and the accuracy of predictive models (Bolnick et al., 2003). For example, in a population of bluegill sunfish (*Lepomis macrochirus*), prior experience foraging on a single prey type increases the likelihood of an individual using that resource, even when another resource becomes more profitable (Werner, Mittelbach, & Hall, 1981). Theoretical models of predator–prey systems are also much more likely to have chaotic dynamics when the predator lags in switching preferred prey (i.e., is highly specialized) when there are high rates of changes in prey populations (Abrams & Matsuda, 2004). Further, dynamics such as prey density relative to one another and indirect interactions between prey can be sensitive to small variations in the speed of predators changing prey preferences (Abrams & Matsuda, 2004), which would be affected by the predator's level of specialization. More generally, diversification within a population can have significant impacts on communities and the ecosystem, such as prey community structure and total primary production (Harmon et al., 2009). In addition, differences in a single species’ population structure (i.e., feeding diversity) can have larger impacts on community composition than differences between species (Rudolf & Rasmussen, 2013). These findings imply that differences between individuals of the same species can be important drivers of ecosystem functions (Harmon et al., 2009; Rudolf & Rasmussen, 2013). Thus, including metrics of variation in foraging decisions between individuals of the same population in ecosystem studies provides a more clear and accurate description of the system and ignoring them can be an oversimplification of the ecological interactions in the community (Araújo, Bolnick, & Layman, 2011; Bolnick et al., 2003, 2011; Dall, Bell, Bolnick, & Ratnieks, 2012). Unfortunately, most foraging studies do not describe the level of intraspecific variation in the predator population.

Variation in foraging decisions within populations of predators is difficult to describe empirically because it requires observing many predation events in multiple individuals across numerous ecological contexts. The empirical problems are even greater when studying predators that forage in environments where it is difficult to directly observe predation events (e.g., marine environments) and that prey on a large diversity of taxonomically similar prey species that make it difficult to determine which species has been consumed. Here, we demonstrate how the application of an individual diet specialization metric to a large set of molecular prey barcoding data from scat can be used to describe intrapopulation feeding diversity by examining the short‐term variation in individual foraging decisions in a marine predator. Our analysis allowed us to explore (a) correlations of individual diet diversity with the sex of the predator and month in which the predation occurred, and (b) to test whether short‐term diet diversity was related to the consumption of particular prey species and their preferred habitat.

The harbor seal (*Phoca vitulina*) is a well‐studied species with potentially large impacts on its ecosystem. Harbor seals have the largest worldwide distribution of any pinniped in coastal areas (Teilmann & Galatius, 2018), are an abundant marine predator in the Salish Sea (Jeffries, Huber, Calambokidis, & Laake, 2003; Olesiuk, 2009), and appear to have reached carrying capacity in that region (Jeffries et al., 2003; Olesiuk, 2009). Because harbor seals are abundant in the ecosystem and feed on a wide range of species, they have significant impacts on prey populations (Howard, Lance, Jeffries, & Acevedo‐Gutiérrez, 2013; Lance, Chang, Jeffries, Pearson, & Acevedo‐Gutiérrez, 2012; Olesiuk, Bigg, Ellis, Crockford, & Wigen, 1990). Some of their prey species are of high conservation concern, such as Pacific salmon (*Oncorhynchus* spp.; hereafter referred to as Salmoniformes), rockfish (*Sebastes* spp.), and Pacific herring (*Clupea pallasii pallasii*, Valenciennes, 1847) (Bjorkland et al., 2015; Bromaghin et al., 2013; Lance et al., 2012). There is special interest surrounding their impact on Chinook salmon (*Oncorhynchus tshawytscha*) as their consumption of this species appears to have increased over the last few decades (Adams et al., 2016; Chasco et al., 2017). Chinook salmon are of special concern given their cultural and economic importance in the Pacific Northwest, and their role as prey for another population of concern: southern resident killer whales (*Orcinus orca*) (Ford, Ellis, Olesiuk, & Balcomb, 2010; Hanson et al., 2010). Harbor seal's effect on Salmoniformes is also of interest because they eat both juvenile and adult individuals (Thomas, Nelson, Lance, Deagle, & Trites, 2017). Eating juveniles may have considerable impacts on populations of Chinook, coho (*Oncorhynchus kisutch*) and steelhead (*O. mykiss*), as survival during the first several months at sea is believed to be the primary factor limiting population abundance and productivity (Beamish et al., 2010; Kendall, Marston, & Klungle, 2017; Neville, Beamish, & Chittenden, 2015).

Due to the large diversity of prey species that harbor seal populations eat, the species has historically been considered a generalist predator (Teilmann & Galatius, 2018). However, several lines of evidence support the notion that harbor seal populations in the Salish Sea may instead be comprised of individual specialists (Bjorkland et al., 2015; Schwarz et al., 2018). They are viewed as central place foragers due to their high haul‐out site fidelity (Peterson, Lance, Jeffries, & Acevedo‐Gutiérrez, 2012; Suryan & Harvey, 1988). Central place foraging, in combination with harbor seal's high abundance in the region (Jeffries et al., 2003; Olesiuk, 2009), makes high intraspecific competition likely, which in turn increases the likelihood of individual specialization (Araújo et al., 2011). Because harbor seals impact prey populations of conservation concern, it is important to examine the prevalence of this potential specialization to better understand the dynamics between seals and their prey.

Prey composition and foraging dive behavior of harbor seals in the Salish Sea vary relative to habitat, sex, and time of year (Lance et al., 2012; Olesiuk et al., 1990; Wilson, Lance, Jeffries, & Acevedo‐Gutiérrez, 2014). Harbor seals also eat different types of prey depending on the type of environment in which they forage. Scat samples from haul‐outs located in estuaries have higher prey diversity than those coming from outside estuaries (Lance et al., 2012; Luxa & Acevedo‐Gutiérrez, 2013). Further, males and females consume different prey (Bjorkland et al., 2015; Schwarz et al., 2018) and have different foraging dive patterns (Wilson et al., 2014). Specifically, females frequently perform longer and deeper foraging dives than males, and more commonly consume benthic species (Schwarz et al., 2018; Wilson et al., 2014). Harbor seal behavioral differences between seasons and sexes exist outside of the Salish Sea as well. Females in Scotland spend more time at sea after lactation, indicative of increased foraging, while males increase their time spent hauling out during the molt, indicative of decreased foraging (Thompson, Fedak, McConnell, & Nicholas, 1989).

These traits of high abundance and differences in diet and foraging patterns between males and females suggest that harbor seals could display intrapopulation feeding diversity. As such, ecosystem dynamics, with regard to the effect of harbor seals on prey species, are likely more complex than described in current models of the system, which assume consistent generalized behavior (e.g., Chasco et al., 2017; Howard et al., 2013). As harbor seals are the most abundant mammalian predator in the Salish Sea and prey on many species of economic and conservation concern, an accurate understanding of their role in ecosystem dynamics is important, for which high quality diet data are required. While current bioenergetics‐based models are useful descriptors of harbor seal consumption (e.g., Chasco et al., 2017; Howard et al., 2013), they can be improved by including the effects of different foraging strategies across sexes and individuals.

Obtaining high quality diet data from large, mobile organisms, such as marine mammals, can be costly and time‐consuming (Rothstein, McLaughlin, Acevedo‐Gutiérrez, & Schwarz, 2017). Analysis of prey contents in scat via metabarcoding is a relatively cheap, noninvasive, and time‐efficient way to obtain large sample sizes with species‐level taxonomic resolution (Deagle et al., 2005, 2019; Rothstein et al., 2017; Tollit et al., 2009). However, to our knowledge, these molecular techniques have not previously been used to quantify intrapopulation feeding diversity at large spatial and temporal scales.

Measuring long‐term individual specialization via longitudinal studies is logistically complicated, expensive, and invasive when studying large, mobile, wild animals, particularly marine mammals. Consequently, individual specialization studies of marine mammals tend to be limited to single years and relatively few collection sites (≤3) and samples (≤100) (e.g., Kernaléguen, Arnould, Guinet, & Cherel, 2015; Rita, Drago, Galimberti, & Cardona, 2017; Rossman et al., 2015). Cross‐sectional sampling is an alternative approach that circumvents these issues because it only requires measurements from a single time point. While cross‐sectional data can provide estimates of long‐term individual specialization (Beaudoin, Tonn, Prepas, & Wassenaar, 1999; Gu, Schelske, & Hoyer, 1997), there are limitations to cross‐sectional sampling as perceived specialization decreases with increased time over which observations are pooled (Novak & Tinker, 2015). Thus, the timeframe over which diet data are collected can influence resulting calculations and limit interpretation. Yet, comparisons of individual specialization metrics calculated from cross‐sectional data collected in the same manner and over the same timeframe will provide an accurate description of short‐term intrapopulation feeding diversity.

Here, we use molecular barcoding of prey DNA from scat in a novel way to examine intrapopulation feeding diversity in harbor seals by answering the following questions: (a) How do the factors Sex, Month, Location, and Year affect cross‐sectional estimation of a specialization metric? (b) What prey items correlate with high levels of specialization relative to sex and environment? To answer these questions, we collected and analyzed scat from wild harbor seals in the Salish Sea. Diet of harbor seals was determined from the scat using molecular techniques and supplemented with hard‐part techniques to determine the proportion of juvenile and adult Salmoniformes consumed. Sex of the depositor was determined using molecular techniques. Diet data were analyzed with a proportional similarity index (Bolnick, Yang, Fordyce, Davis, & Svanbäck, 2002) to describe the variation in individual foraging decisions in harbor seals.

## 2 METHODS

### 2.1 Collection and processing of scat samples

**Figure 1:**
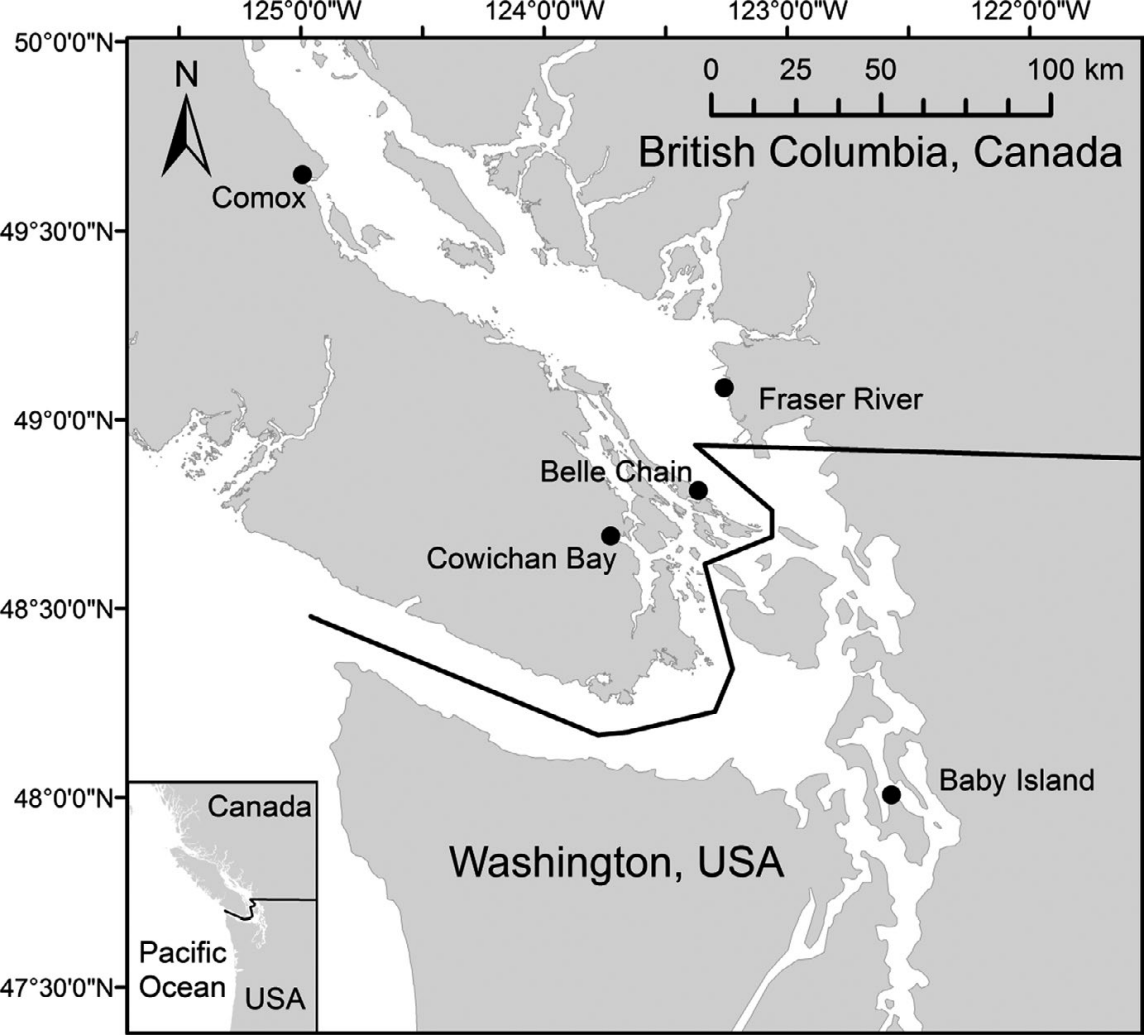
Haul‐out sites where harbor seal scat was collected in the Salish Sea. Collection locations are indicated by black dots and labeled with the name used throughout this paper. Coordinates for each site are as follows: Comox: 49°35'45.53"N, 124°52'4.39"W, Fraser River: 49° 4'27.17"N, 123° 8'49.46"W, Belle Chain: 48°58'10.73"N, 123°29'34.63"W, Cowichan Bay: 48°44'14.28"N, 123°37'17.76"W, Baby Island: 48° 5'58.31"N, 122°31'41.29"W

Scat collections were conducted by multiple researcher groups at five harbor seal haul‐outs (areas were seals congregate out of water) in the Salish Sea over a period of four nonsequential years (Figure 1). Haul‐outs varied in seal population size as well as by habitat type (Table 1). Not all sites were visited every year and the months during which each site was visited varied between years (Table 1). Collections at Belle Chain, Cowichan, Comox, and Fraser River were conducted by teams from University of British Columbia under Fisheries and Oceans Canada's Marine Mammal Research Licenses (MML 2011‐10 and MML 2014‐07) and University of British Columbia's Animal Care Permits (A11‐0072 and A14‐0068) awarded to University of British Columbia Marine Mammal Research Unit. Collections at Baby Island were conducted by a team from Western Washington University under Federal Permit 18,002 from the United States Office of Protected Resources, National Marine Fisheries Service, and a Western Washington University's Animal Care and Use Committee exception awarded to Alejandro Acevedo‐Gutiérrez.

**Table 1:** Groups for analysis of specialization in harbor seals in the Salish Sea

Location	Month	Year	Season	Sex	Minimum prey density	Theoretical minimum	*N*	SI	Use	Shannon–Weaver
BC	June	2012	Summer	Male	0.0110	0.2000	5	0.549	Yes	0.928
BC	June	2012	Summer	Female	0.0160	0.5000	2	0.680	No	1.236
BC	July	2012	Summer	Male	0.0002	0.1111	9	0.380	Yes	1.935
BC	July	2012	Summer	Female	0.2968	1.0000	1	0.000	No	0.608
BC	Aug	2012	Summer	Female	0.0001	0.0556	18	0.421	Yes	1.567
BC	Aug	2012	Summer	Male	0.0002	0.0588	17	0.390	Yes	1.618
BC	Sept	2012	Fall	Female	0.0124	0.0526	19	0.572	Yes	0.987
BC	Sept	2012	Fall	Male	0.0076	0.0833	12	0.427	Yes	1.600
BC	Aug	2013	Summer	Female	0.0139	0.2000	5	0.314	Yes	2.121
BC	Aug	2013	Summer	Male	0.0114	0.2000	5	0.478	Yes	1.145
BC	Sept	2013	Fall	Female	0.0103	0.0714	14	0.527	Yes	1.514
BC	Sept	2013	Fall	Male	0.0105	0.0769	13	0.487	Yes	1.403
BC	Oct	2013	Fall	Male	0.0107	0.0833	12	0.588	Yes	1.144
BC	Oct	2013	Fall	Female	0.0104	0.3333	3	0.614	No	0.884
BI	Apr	2016	Spring	Female	0.0120	0.1429	7	0.426	Yes	1.754
BI	Apr	2016	Spring	Male	0.0297	0.1429	7	0.387	Yes	1.169
BI	May	2016	Spring	Male	0.0094	0.0278	36	0.387	Yes	1.587
BI	May	2016	Spring	Female	0.0118	0.0370	27	0.305	Yes	1.751
BI	June	2016	Summer	Male	0.0122	0.0833	12	0.246	Yes	2.123
BI	June	2016	Summer	Female	0.0251	0.1250	8	0.214	Yes	1.942
BI	July	2016	Summer	Female	0.0477	0.1250	8	0.341	Yes	1.193
BI	July	2016	Summer	Male	0.2368	0.3333	3	0.529	No	0.879
CB	June	2012	Summer	Female	0.0006	0.1111	9	0.474	Yes	1.634
CB	June	2012	Summer	Male	0.0002	0.1429	7	0.344	Yes	1.717
CB	July	2012	Summer	Male	0.0003	0.0500	20	0.447	Yes	1.557
CB	July	2012	Summer	Female	0.0034	0.0625	16	0.395	Yes	2.115
CB	Aug	2012	Summer	Female	0.0002	0.1429	7	0.316	Yes	1.556
CB	Sept	2012	Fall	Female	0.0007	0.0526	19	0.313	Yes	1.846
CB	Sept	2012	Fall	Male	0.0029	0.1429	7	0.330	Yes	1.580
CB	Oct	2012	Fall	Male	0.0002	0.0625	16	0.263	Yes	2.059
CB	Oct	2012	Fall	Female	0.0004	0.0769	13	0.320	Yes	1.745
CB	Nov	2012	Fall	Male	0.0137	0.2500	4	0.988	No	0.063
CB	Nov	2012	Fall	Female	0.0110	0.5000	2	0.995	No	0.034
CB	Apr	2013	Spring	Female	0.0278	0.1000	10	0.352	Yes	1.705
CB	Apr	2013	Spring	Male	0.1160	1.0000	1	0.000	No	0.359
CB	May	2013	Spring	Female	0.0112	0.0909	11	0.313	Yes	1.979
CB	May	2013	Spring	Male	0.0123	0.2500	4	0.374	No	1.059
CB	June	2013	Summer	Female	0.0167	0.1111	9	0.489	Yes	1.337
CB	June	2013	Summer	Male	0.0154	0.3333	3	0.337	No	1.328
CB	July	2013	Summer	Female	0.0114	0.1250	8	0.400	Yes	1.610
CB	July	2013	Summer	Male	0.0116	0.1429	7	0.384	Yes	1.728
CB	Aug	2013	Summer	Female	0.0103	0.0667	15	0.228	Yes	2.147
CB	Aug	2013	Summer	Male	0.0143	0.2500	4	0.393	No	1.256
CB	Sept	2013	Fall	Female	0.0187	0.0833	12	0.327	Yes	1.687
CB	Sept	2013	Fall	Male	0.0108	0.1000	10	0.354	Yes	1.959
CB	Oct	2013	Fall	Female	0.0122	0.0833	12	0.546	Yes	1.313
CB	Oct	2013	Fall	Male	0.0103	0.1111	9	0.463	Yes	1.362
CB	Nov	2013	Fall	Male	0.0116	0.1250	8	0.531	Yes	0.932
CB	Nov	2013	Fall	Female	0.0110	0.1429	7	0.486	Yes	1.317
CB	May	2014	Spring	Male	0.0047	0.1000	10	0.222	Yes	1.911
CB	May	2014	Spring	Female	0.0126	0.1667	6	0.412	Yes	1.495
CB	June	2014	Summer	Male	0.0113	0.1250	8	0.365	Yes	1.509
CB	June	2014	Summer	Female	0.0105	0.1667	6	0.349	Yes	1.542
CB	July	2014	Summer	Male	1.0000	1.0000	1	0.000	No	0.000
CB	Aug	2014	Summer	Male	0.0008	0.3333	3	0.341	No	1.572
CB	Aug	2014	Summer	Female	0.0200	0.5000	2	0.600	No	1.292
CB	Sept	2014	Fall	Male	0.0139	0.1111	9	0.256	Yes	1.729
CB	Sept	2014	Fall	Female	0.0007	0.1250	8	0.601	Yes	1.127
CB	Oct	2014	Fall	Male	0.0024	0.0270	37	0.265	Yes	2.048
CB	Oct	2014	Fall	Female	0.0104	0.0417	24	0.225	Yes	2.230
CB	Nov	2014	Fall	Male	0.0003	0.0435	23	0.249	Yes	1.820
CB	Nov	2014	Fall	Female	1.0000	1.0000	1	0.000	No	0.000
CM	May	2012	Spring	Male	0.0001	0.0667	15	0.570	Yes	1.568
CM	May	2012	Spring	Female	0.0144	0.2500	4	0.586	No	0.993
CM	June	2012	Summer	Male	0.0017	0.0556	18	0.295	Yes	2.150
CM	June	2012	Summer	Female	0.0007	0.0909	11	0.338	Yes	2.362
CM	July	2012	Summer	Male	0.0004	0.0455	22	0.269	Yes	2.357
CM	Aug	2012	Summer	Male	0.0012	0.0909	11	0.272	Yes	1.945
CM	Aug	2012	Summer	Female	0.0008	0.1111	9	0.232	Yes	2.216
CM	Sept	2012	Fall	Female	0.0105	0.0500	20	0.240	Yes	2.200
CM	Sept	2012	Fall	Male	0.0019	0.1000	10	0.268	Yes	1.986
CM	Oct	2012	Fall	Female	0.0000	0.0625	16	0.251	Yes	1.814
CM	Oct	2012	Fall	Male	0.0000	0.1250	8	0.575	Yes	0.691
CM	Apr	2013	Spring	Male	0.0015	0.0769	13	0.317	Yes	1.560
CM	Apr	2013	Spring	Female	0.0120	0.2500	4	0.304	No	1.761
CM	May	2013	Spring	Female	0.0116	0.0833	12	0.213	Yes	2.219
CM	May	2013	Spring	Male	0.0144	0.2000	5	0.292	Yes	1.334
CM	June	2013	Summer	Male	0.0105	0.1250	8	0.206	Yes	1.901
CM	June	2013	Summer	Female	0.0179	0.2000	5	0.361	Yes	1.455
CM	July	2013	Summer	Male	0.0135	0.0909	11	0.600	Yes	1.203
CM	July	2013	Summer	Female	0.0220	0.1429	7	0.540	Yes	0.985
CM	Aug	2013	Summer	Female	0.0135	0.1000	10	0.275	Yes	2.141
CM	Aug	2013	Summer	Male	0.0125	0.1000	10	0.423	Yes	1.615
CM	Sept	2013	Fall	Female	0.0112	0.0625	16	0.256	Yes	1.995
CM	Sept	2013	Fall	Male	0.0133	0.1111	9	0.506	Yes	1.296
CM	Oct	2013	Fall	Female	0.0120	0.0909	11	0.328	Yes	1.778
CM	Oct	2013	Fall	Male	0.0111	0.1429	7	0.463	Yes	1.333
FR	May	2012	Spring	Male	0.0025	0.0769	13	0.302	Yes	2.388
FR	June	2012	Summer	Male	0.0055	0.0588	17	0.372	Yes	2.479
FR	June	2012	Summer	Female	0.0005	0.3333	3	0.574	No	1.865
FR	July	2012	Summer	Male	0.0101	0.0476	21	0.771	Yes	0.728
FR	July	2012	Summer	Female	0.0118	0.5000	2	0.585	No	1.341
FR	Aug	2012	Summer	Male	0.0105	0.1667	6	0.974	Yes	0.127
FR	Aug	2012	Summer	Female	0.0130	0.5000	2	0.976	No	0.140
FR	Sept	2012	Fall	Male	0.0102	0.0345	29	0.388	Yes	1.310
FR	Sept	2012	Fall	Female	0.0016	0.0769	13	0.206	Yes	2.020
FR	Oct	2012	Fall	Male	0.0110	0.0769	13	0.976	Yes	0.092
FR	Oct	2012	Fall	Female	0.0124	0.2000	5	0.648	Yes	0.701
FR	Apr	2013	Spring	Male	0.0110	0.1111	9	0.523	Yes	0.955
FR	Apr	2013	Spring	Female	0.0108	0.2000	5	0.463	Yes	1.190
FR	May	2013	Spring	Male	0.0152	0.0667	15	0.246	Yes	2.325
FR	May	2013	Spring	Female	0.0123	0.2500	4	0.373	No	1.413
FR	June	2013	Summer	Male	0.0135	0.3333	3	0.361	No	1.623
FR	June	2013	Summer	Female	0.0107	1.0000	1	0.000	No	0.059
FR	Aug	2013	Summer	Male	1.0000	0.5000	2	1.000	No	0.000
FR	July	2013	Summer	Male	0.0118	0.2000	5	0.269	Yes	1.808
FR	July	2013	Summer	Female	0.0455	1.0000	1	0.000	No	1.679
FR	Aug	2013	Summer	Female	0.0119	0.3333	3	0.420	No	1.483
FR	Sept	2013	Fall	Female	0.0112	0.1429	7	0.811	Yes	0.626
FR	Oct	2013	Fall	Male	0.0105	0.0833	12	0.772	Yes	0.731
FR	Oct	2013	Fall	Female	0.0047	0.2000	5	0.562	Yes	1.316

Note

BC = Belle Chain, BI = Baby Island, CB = Cowichan Bay, CM = Comox, FR = Fraser River.

Location denotes where the group of scat was collected. Month, Year, and Season show when the group of scat was collected. The minimum prey density column indicates the lowest occurring prey proportion within a single scat within the group. The theoretical minimum column indicates the theoretical minimum $PS_{i}$ that was assigned to each group. The theoretical minimum was calculated by dividing one by the total sample size of each group. *N* indicates the total number of samples within each group. SI indicates the average $PS_{i}$ within each group. The use column indicates whether or not the group was used in downstream analysis based off of sample size (groups with < 5 samples were excluded). The Shannon–Weaver column indicates the Shannon–Weaver index assigned to each group. This index was calculated by averaging prey proportions from each scat within each group and subsequent use of the diversity function in the VEGAN package (Oksanen et al., 2018) in R 3.3.1.

Collection of scat followed the general procedure described in Thomas, Deagle, Eveson, Harsch, and Trites (2016). Briefly, upon arrival at a haul‐out, we searched the entire area for scat. When scat was found, the entire scat was collected into a 126‐μm nylon strainer inside of a 500‐ml sealable container using a wooden tongue dispenser and plastic spoon. The container was then stored in a cooler with ice until transfer to a −20°C freezer later that day. At Baby Island and Cowichan Bay in 2014, the entire outside of the scat was swabbed before collection. Swabbing focused on any mucus material, as it likely contains higher proportions of seal DNA (Rothstein, 2015). The swab was then placed in a vial of ethanol and stored in a cooler with ice until transfer to a −20°C freezer later that day.

A DNA slurry of homogenized scat in ethanol was prepared for each sample to obtain a representative set of DNA following the procedure described in Thomas et al. (2016). Briefly, the entire scat was thawed in ethanol and homogenized within the mesh bag. After homogenization, a representative sample of DNA slurry was allowed to pass through the bag. The mesh bag was then removed, zip‐tied, and stored at −20°C for later use in prey hard‐part (i.e., bones, otoliths) analysis. We then let the DNA slurry settle in the containers on the bench top overnight. The next day we pipetted the settled slurry into 20‐ml scintillation vials that were subsequently stored at −20°C until further analysis.

### 2.2 Sex determination of harbor seals via scat

To obtain DNA for sex determination, DNA was extracted from the scat matrix‐ethanol slurry for all locations, except Cowichan 2014 and Baby Island. For these last two sites, DNA was extracted from the swabs. To extract DNA from swabs, the excess ethanol from the vial was poured off and the swab was dried in a vacuum centrifuge at 39°C until all ethanol had evaporated, approximately one hour. We then used QIAGEN DNeasy Blood and Tissue Kit to extract DNA from the dried swabs. DNA was extracted from slurry matrixes using QIAGEN QIAamp DNA Stool Mini Kit. Extracted DNA, from either the ethanol slurry or swab, was used in Taqman quantitative polymerase chain reactions (qPCR) to determine the presence and absence of X and Y chromosomes. The procedure was modified from Matejusová et al. (2013) and is described in depth in Rothstein (2015) and Schwarz et al. (2018). The two probes that we used targeted the paralogous zinc finger X (ZFX) and zinc finger Y (ZFY) genes. All primers and probes are described in Matejusavá et al. (2013) and are as follows: ZFX; F: AGAGCAACCCTGTCATAAAGAGAAC, R: GGACTGAGGTTGGTACAATCAGACT, P: 6FAM‐CTGGTCTGAAAACTTCATT‐MGB.

ZFY; F: GCAAGCTCCGAGATTAAACCA, R: TGATCTAGCAGCTAAATTGCTATCG, P: 6FAM‐TGTACCCACAGAGGTGT‐MGB. Two reactions were run for each sample with each probe (four reactions total per sample). Each reaction consisted of 4.5 μl of ABI Taqman gene expression master mix, 0.5 μl of either the ZFX or ZFY probe, and 5 μl of DNA template. Reactions were run on a quantitative thermocycler with the following protocol: one holding cycle (50°C for 2 min, 95°C for 10 min) followed by 60 cycles of denaturation and annealing/extension (95°C for 15 s, 60°C for 1 min). Four positive (two reactions for each sex, one ZFX, and one ZFY probe each) and four negative controls (two reactions for each ZFX and ZFY probe) were run with each set of reactions. Positive controls came from captive harbor seals of known sex at the Vancouver Aquarium in Vancouver, BC, and Point Defiance Zoo & Aquarium in Tacoma, WA. Negative controls consisted of PCR grade water in place of a DNA template.

If no amplification occurred in either ZFX reactions, the sample was excluded from further analysis. We tallied a sample as being deposited by a male if amplification was observed in any of both reactions with the ZFY probe and in any of both reactions with the ZFX probe. We tallied a sample as being deposited by a female if amplification occurred in any of both ZFX reactions, but no amplification occurred in any ZFY reaction. The false‐negative rate for two failed ZFY reactions (and thereby incorrectly classifying a male as a female) was 1.35%. This value was calculated from the occurrence of only one of the two ZFY reactions having positive amplification within a sample that was classified as male for all samples tested in this study. Although this false‐negative rate is low, we excluded any samples that amplified in ZFY reactions but failed to amplify in ZFX reactions.

### 2.3 Prey determination in harbor seal scat

The diet of harbor seals was determined by combining DNA and hard‐part data. The DNA prey identification and quantification were completed following the procedure outlined in Thomas et al. (2016). Briefly, for all locations, the scat matrix DNA (obtained from extracting DNA out of the DNA slurry using QIAGEN QIAamp DNA Stool Mini Kit) for each sample underwent a multiplex PCR using primers for a 16S mtDNA barcoding fragment (~260 bp) described by Deagle, Chiaradia, McInnes, and Jarman (2010). These primers sets were designed to amplify chordate and cephalopod sequences and are as follows: Chord 16S *F* (GATCGAGAAGACCCTRTGGAGCT), Chord 16S R (GGATTGCGCTGTTATCCCT), Ceph_ 16S *F* (GACGAGAAGACCCTAWTGAGCT), and Ceph 16S R (AAATTACGCTGTTATCCCT) (Deagle et al., 2010; Thomas et al., 2016). Amplicons were labeled using a combination of unique F and R primer tags, in addition to indexed, post‐PCR ligated Illumina TruSeq™ adapter sequences (for details see Thomas et al., 2016). An Illumina MiSeq was then used to sequence the amplified DNA fragments. Lastly, a custom BLAST database comprised of publicly available reference sequences specific for known prey species was used to produce identifications to the lowest taxonomic level possible for each amplified sequence.

Extraction and preparation of prey hard parts were completed by Thomas et al. (2017) for Belle Chain, Comox, Cowichan Bay, and Fraser River 2012 and 2013 samples, by one of the authors (BAN) for Cowichan Bay 2014, and by the first author (MRV) for Baby Island samples. Each scat was placed in a set of nested sieves and then rinsed and stirred until all that was left in the sieves were prey hard parts. All hard parts, except cephalopod beaks, were transferred to 20‐ml scintillation vials with 70% ethanol. They were allowed to sit for a minimum of two weeks before the liquid was poured off and the hard parts were allowed to dry. The cephalopod beaks were transferred to separate 20‐ml scintillation vials with ethanol. All diagnostic prey hard parts were identified to the lowest taxonomic level possible using reference sets of prey bones from Washington and British Columbia by Thomas et al. (2017) for Belle Chain, Comox, Cowichan Bay, and Fraser River and by collaborators at Long Live The Kings for Baby Island samples. Published keys for both fish bones and cephalopod beaks were used as described in Thomas et al. (2017). Notably, this analysis allowed differentiation between the proportion of adult and juvenile Salmoniformes consumed. The percentage of juvenile versus adult salmon was determined using the method described in Thomas et al. (2017). Briefly, the DNA percentages assigned to each salmon species were split based on the ratio of juvenile versus adult salmon documented during hard‐part analysis. The ratio was determined using data from either the sample, month, or season, dependent on available data. This information was used to investigate whether juvenile and adult salmon were being consumed differently with respect to specialization habits (addressed in Section 2.6). This additional analysis was performed due to the high economic and environmental impact salmon species have in the region and previous evidence that harbor seals have a significant impact on salmon populations at the juvenile life stage (Thomas et al., 2017).

### 2.4 Quantification of specialization metrics

Cross‐sectional sampling, which only requires data from a single time point, can be used to estimate intrapopulation feeding diversity at large spatial and temporal scales. For pinnipeds, a single time point can be examined via scat collection and analysis with a single scat being indicative of the last few foraging bouts (Bowen & Iverson, 2012). Cross‐sectional diet studies assume that each sample consists of multiple prey items and that each item represents an independent capture decision (Araújo et al., 2011). It is also assumed that the sampled diet is representative of the complete diet of the individual (Araújo et al., 2011). If both assumptions are met cross‐sectional samples allow for estimates of true individual specialization. However, it is unlikely these assumptions are always met for pinnipeds as their prey can be clumped (i.e., forage fish), which would result in one foraging decision influencing the next. Further, it is unlikely that a scat represents the entire diet of an individual as a single scat is only indicative of the last few foraging bouts (Bowen & Iverson, 2012). In these cases, one cannot calculate absolute individual specialization using cross‐sectional sampling (Araújo et al., 2011; Novak & Tinker, 2015). However, one can compare relative specialization between samples collected in the same manner (i.e., within the dataset). Thus, by calculating specialization metrics based on prey proportions in harbor seal scat, we were able to deepen our understanding of intrapopulation feeding diversity and uncover patterns at the level of sex, month, and location.

To this end, we quantified the level of specialization represented by each sample using the proportional similarity index ($PS_{i}$) function in the R package RInSp (Zaccarelli, Bolnick, & Mancinelli, 2013). $PS_{i}$ calculates the overlap between what an individual is eating and what the population is eating using the following formula:

$$PS_{i}=1‐0.5\sum_{j}\left|p_{\mathrm{ij}}‐q_{j}\right|$$

where *p_ij_* represents the proportion of resource *j* used by the individual *i*, and *q_j_* represents the proportion of resource *j* used by the population. $PS_{i}$ is bounded by a theoretical minimum, which is population dependent as described below, and one. The variable population‐dependent minimum indicates a complete specialist and a $PS_{i}$ of one indicates a generalist (Bolnick et al., 2002). Because $PS_{i}$ is bounded, we report the overall average value with 95% confidence intervals calculated using Monte Carlo resampling in the R 3.3.1 package “resample.” Traditionally, prey counts have been used for calculating $PS_{i}$, not proportions, as each count is assumed to represent an independent prey capture decision (Araújo et al., 2011; Bolnick et al., 2002). Proportions of prey metabarcoding reads are representations of the prey biomass proportions that were consumed by the predator, and similar proportions can result from consuming a few large or many small prey individuals. Correspondingly, calculating $PS_{i}$, using the proportions of prey metabarcoding reads, will produce a metric of “biomass specialization” that does not necessarily reflect independent prey capture decisions. Nevertheless, it describes intrapopulation variations in the utilization of different prey species. In addition, we calculated $PS_{i}$ relative to groups of samples from a certain point in space and time. If individuals in that particular group are encountering the same size distribution of prey, then diet proportions may represent the same relative relationship of prey capture decisions as counts of individual prey items. Despite the potential limitations, there are several benefits to using this type of data. Coupled with scat collection, it allows for large sample sizes, is noninvasive, and gives high taxonomic resolution.

To define our groups for analysis, samples were separated by Location, Sex, Year, and Month of collection, yielding a total of 111 groups (Table 1). $PS_{i}$ values for each sample were then calculated for each one of these groups. Within each group, each sample was treated as coming from a different individual due to the low probability of resampling the same individual (Rothstein et al., 2017).

Because different groups for analysis can have different theoretical minima, there is potential bias when comparing specialization metrics across groups. Differences in theoretical minima occur due to differences in sample size (the number of scat in each group) and/or differences in minimum prey densities (the smallest occurring proportion of a prey species in a group's diet). Due to very low minimum prey densities in our data set, the theoretical minima are determined by sample size (Table 1). We addressed this potential bias in multiple ways. First, we excluded from analysis the smallest groups (those with < 5 samples) as they have the highest theoretical minimum and thus the most potential for bias. We also used Spearman's rank correlation to estimate how much variance was explained by differences in sample size. This correlation was accomplished by comparing sample size to the average $PS_{i}$ for each group we kept. We also calculated the theoretical minima for each group by dividing one by the number of samples in the group and then examined the range, average, and median of those minima. Additionally, sample sizes of each group were included in modeling of the data, which is described below. Lastly, the seasonal changes in sample size were visually compared with the seasonal patterns in $PS_{i}$ values.

### 2.5 Comparison of factors influencing relative specialization

We analyzed the relative influence of the factors Sex, Month, Location, Year, and Sample Size on $PS_{i}$ using generalized linear mixed models (GLMMs). We chose mixed models because they allowed us to include Sample Size, Location, and Year as random variables. Restricted maximum‐likelihood estimation was used because it considers the loss of degrees of freedom when estimating fixed effects and thus offers a more unbiased estimate than maximum‐likelihood methods (West, Welch, & Galecki, 2015). Before modeling the data, we performed a logit transformation ($\log(\frac{{\mathrm{PS}}_{i}}{1‐{\mathrm{PS}}_{i}}$)) on the $PS_{i}$ values to normalize them. This transformation was necessary because $PS_{i}$ is bounded by a theoretical minimum and one, which affects the variance distribution (Bolnick et al., 2002; Sokal & Rohlf, 2012). When numbers are bounded, the variance distribution is shifted toward the mean (Sokal & Rohlf, 2012). A logit transformation is an excellent choice for addressing this shift because it extends the tails of the distribution more than other alternatives (Warton & Hui, 2011).

All models were tested in the R 3.3.1 package lme4 (Bates, Mächler, Bolker, & Walker, 2015). This package provides basic measurements of goodness of fit including AIC and coefficients. The R 3.3.1 package MuMIn was used to determine the *r* ^2^ values for mixed models. Subsequent calculations of ∆*AIC* and *w_i_* (positive Akaike weights or likelihood of being the best model (Anderson, 2008)) were completed using Excel. ∆*AIC* was calculated as the difference between two AIC scores; *w_i_* was calculated following Burnham and Anderson (2010).

**Figure 2:**
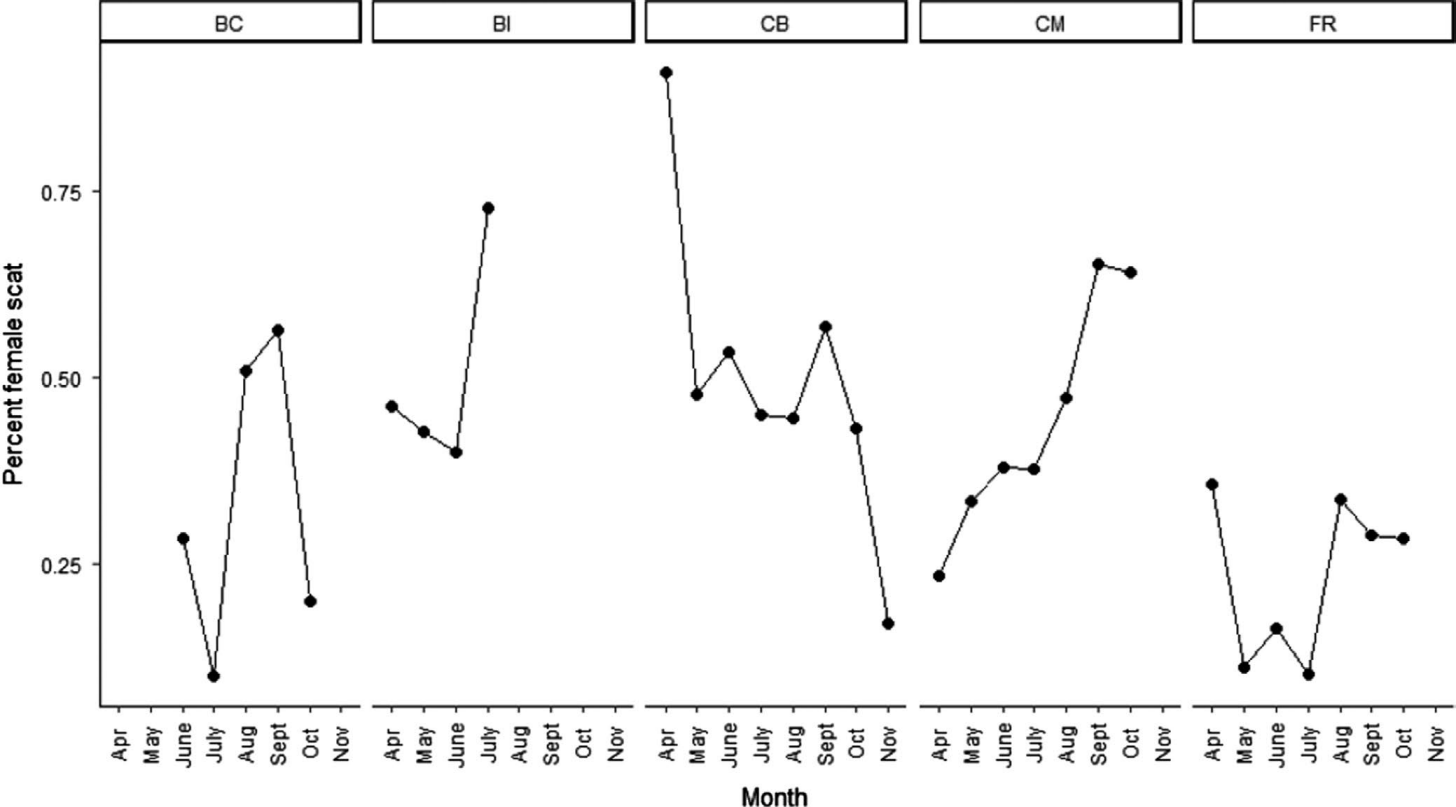
Proportion of female harbor seal scat identified during each month at each site. If no dot is present, no scat was collected at the site in that month. Proportions were calculated by pairing male and female groups for analysis from the same month, location, and year. The number of samples in the female group was divided by the total number of samples in both the female and male group

To understand the relationship between sex ratio of the population and $PS_{i}$, sex ratios were produced for every paired group (groups of males and females from the same location, month, and year) by calculating the percent of scat identified as female. The average $PS_{i}$ for each paired group was then compared with this female percentage using a Spearman's rank correlation. Spearman's rank correlation was used to account for the heteroscedasticity of the dataset and was completed using R 3.3.1. Additionally, the average proportion of female scat for each month and location was visualized (Figure 2).

### 2.6 Correlations between prey items and relative specialization

For each scat, prey item proportions (number of sequences per prey species/ total number of sequences generated) were lumped into orders and summed. To investigate differential impacts on juvenile versus adult salmon, all salmon species where spilt into proportion juvenile and proportion adult based on the hard‐part collection and analysis (see Section 2.3) and summed as two separate orders ("Juvenile Salmoniformes" and "Adult Salmoniformes"), in addition to the Salmoniformes order. We then performed correlations between the proportion of the diet that each order comprised in each sample and the $PS_{i}$ for that sample. To examine whether similar patterns occurred in both sexes, the analysis above was completed for male and females separately. We used the Bonferroni method to correct alpha for multiple comparisons (alpha values are specified in table captions). Additionally, to determine whether benthic species were associated with a more specialist diet, a correlation was run between the proportion benthic prey and specialization value for each scat. Due to the heteroscedasticity of the dataset, we used Spearman's rank method for all correlations (Sokal & Rohlf, 2012). All correlation analysis was conducted in R 3.3.1. Because smaller $PS_{i}$ values indicate higher levels of specialization, a negative correlation value suggests a positive relationship with specialization.

## 3 RESULTS

### 3.1 Quantification of specialization metrics

**Figure 3:**
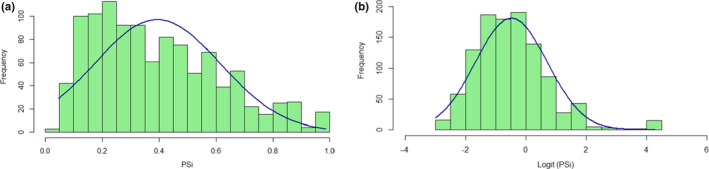
Histogram of $PS_{i}$ values derived from harbor seal scat with kurtosis curve and normal QQ plot for all samples with successful sex determination (*n* = 1,083 scat samples) (a) untransformed $PS_{i}$ and (b) logit‐transformed $PS_{i}$

Over the course of four nonsequential years, at five different locations, we quantified the diet of 1,520 scat samples. The sequence data associated with these samples are stored at: https://figshare.com/s/0113457d4081727aacac. (Thomas et al., in review). We successfully determined the sex of the depositor for 1,145 of those scats (75% success rate). The breakdown of prey proportion within each scat as well as sex assignment for these samples is located on Dryad (10.5061/dryad.59zw3r257). The number of scat with successful sex determination varied by location and month (Table 2). Samples with successful sex determination were then binned by the factors Sex, Location, Month, and Year to form unique groups for analysis (Table 1). After eliminating samples without sex determination and with small sample sizes (<5 samples), we were left with 1,083 samples in 86 groups. Only these 1,083 samples were used in further analyses. $PS_{i}$ was calculated relative to the samples in a specific group, and the average $PS_{i}$ across all samples and groups was 0.399 (95% CI = 0.026, *R* = 100,000). The $PS_{i}$ values of the 1,083 samples were not normally distributed (kurtosis = 2.66, skewness = 0.65, Figure 3). Therefore, a logit transformation was used to adjust the variance distribution (kurtosis = 5.21, skewness = 1.01, Figure 3). These transformed $PS_{i}$ values were used to run the GLMMs. Additionally, the range of theoretical minima across the 86 groups was 0.027–0.2 (average = 0.103, median = 0.091); there was also a correlation between average $PS_{i}$ and theoretical minimum $PS_{i}$ (rho = −0.231, *p* = .03). This potential bias is addressed in the discussion.

**Table 2:** Number of harbor seal scat from the Salish Sea with successful sex determination from all locations, months, and years. Within each monthly column, the numbers are as follows: female scat, male scat. If multiple collection bouts occurred at a single haul‐out within one month, the total number of scat for that month is listed. An “na” indicates no scat were collected at that site during that month. Abundance estimates for Belle Chain, Cowichan Bay, Comox, and Fraser River were calculated from Olesiuk et al. (2009). The abundance estimate for Baby Island was taken from Jeffries et al. (2003)

Location	Environment	Abundance	Year	April	May	June	July	August	September	October	November	Total (by location)
Belle Chain	Rocky reef	834	2012	na	na	2,5	1,9	18,17	19,12	na	na	83
Belle Chain	Rocky reef		2013	na	na	na	na	5,5	14,13	3,12	na	52
Baby Island	Near estuary	<100	2016	7,7	27,36	8,12	8,3	na	na	na	na	108
Cowichan Bay	Estuary	167	2012	na	na	9,7	16,20	7,13	19,7	13,16	2,4	133
Cowichan Bay	Estuary		2013	10,1	11,4	9,3	8,7	15,4	12,10	12,9	7,8	130
Cowichan Bay	Estuary		2014	na	6,10	6,8	0,1	2,3	8,9	24,37	1,23	138
Comox	Near Estuary	121	2012	na	4,15	11,18	13,22	9,11	20,10	16,8	na	157
Comox	Near Estuary		2013	4,13	12,5	5,8	7,11	10,10	16,9	11,7	na	128
Fraser River	Estuary	76	2012	na	0,13	3,17	2,21	2,6	13,29	5,13	na	124
Fraser River	Estuary		2013	5,9	4,15	1,3	1,5	3,2	7,20	5,12	na	92
Total (by month)	na	na	na	56	162	135	155	142	247	203	45	1,145

### 3.2 Comparison of factors influencing relative specialization

**Figure 4:**
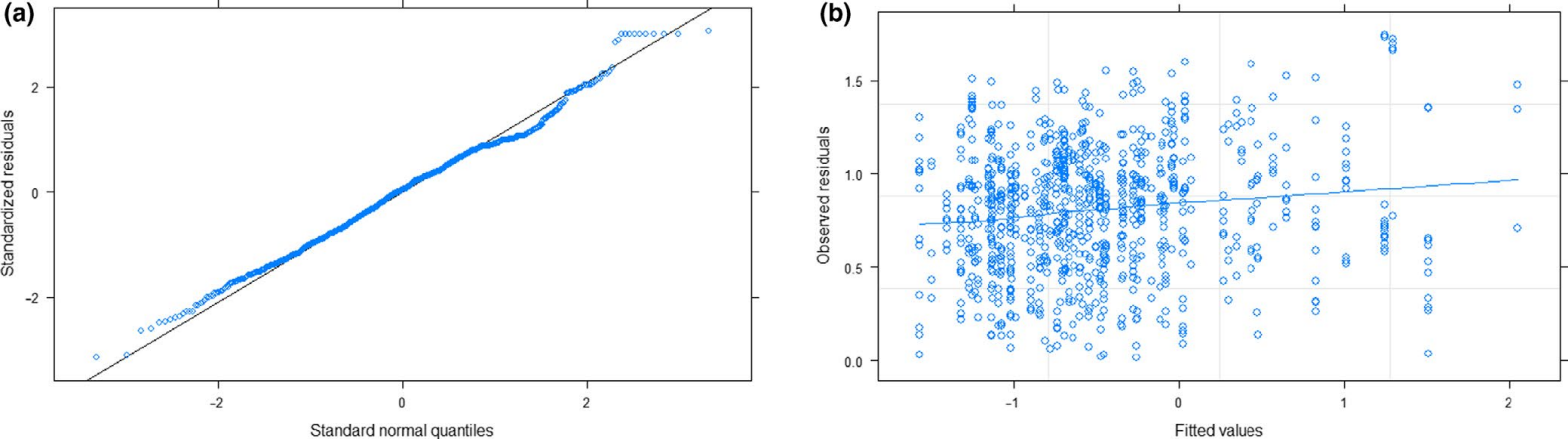
Analysis of fit of the model Sex*Month + (1|Sample Size) + (1|Location) + (1|Year) for harbor seal groups for analysis with >5 samples (*n* = 1,083 scat samples). (a) Standard normal quantiles versus standardized residuals. (b) Fitted values versus observed residuals

Based on AIC values, *r* ^2^ results, and model likelihood, the best fit GLMM was Month*Sex + (1|Sample Size) + (1|Location) + (1|Year) (1| indicates random effects, Table 3). The *r* ^2^ value and residual plots indicate that this model fits the data well (Table 3, Figure 4). The random factors of Sample Size, Location, and Year explained 0.39, 0.36, and 0.002 of the variance (*SD* = 0.62, 0.597, 0.05), respectively. The *r* ^2^ value calculated with fixed and random effects was over four times that of the *r* ^2^ value calculated using just fixed effects. Removing Month from the model caused a larger decrease in goodness‐of‐fit measurements than removing Sex (Table 3). Removal of the interaction term also caused a decrease in goodness‐of‐fit measurements (Table 3). Further, the interaction terms for Sex and the Months of August and October were significant (*t* = 2.86, 2.68, *p* = .004, 0.007, respectively). However, correlation analysis between the percent female scat collected for each paired group (which acted as a proxy for the effect of sex ratio in the population) and the average $PS_{i}$ for that pairing revealed no significant trend (rho = −0.071, *p* = .655).

**Table 3:** GLMM models of prey specialization in Salish Sea harbor seals (1| indicates random effects)

Predictors	r^2^ fixed	r^2^	AIC	$w_{i}$
Sex*Month + (1|Sample size) + (1|Location) + (1|Year)	0.105	0.502	3,141.78	0.99
Sex + Month + (1|Sample size) + (1|Location) + (1|Year)	0.076	0.462	3,157.91	0.03
Month + (1|Sample size) + (1|Location) + (1|Year)	0.061	0.459	3,172.53	2.10E−07
Sex + (1|Sample size) + (1|Location) + (1|Year)	0.017	0.406	3,191.43	1.65E−11

Note

The *r* ^2^ fixed column indicates how much variance was explained solely by the fixed effects. The *r* ^2^ column indicates how much variance was explained by both fixed and random effects. The AIC column indicates the fit of each model; lower values indicate a better model. The $w_{i}$ column indicates the relative likelihood of each model being the best model of those tested. Analysis represents 1,083 samples from groups with > 5 samples.

**Figure 5:**
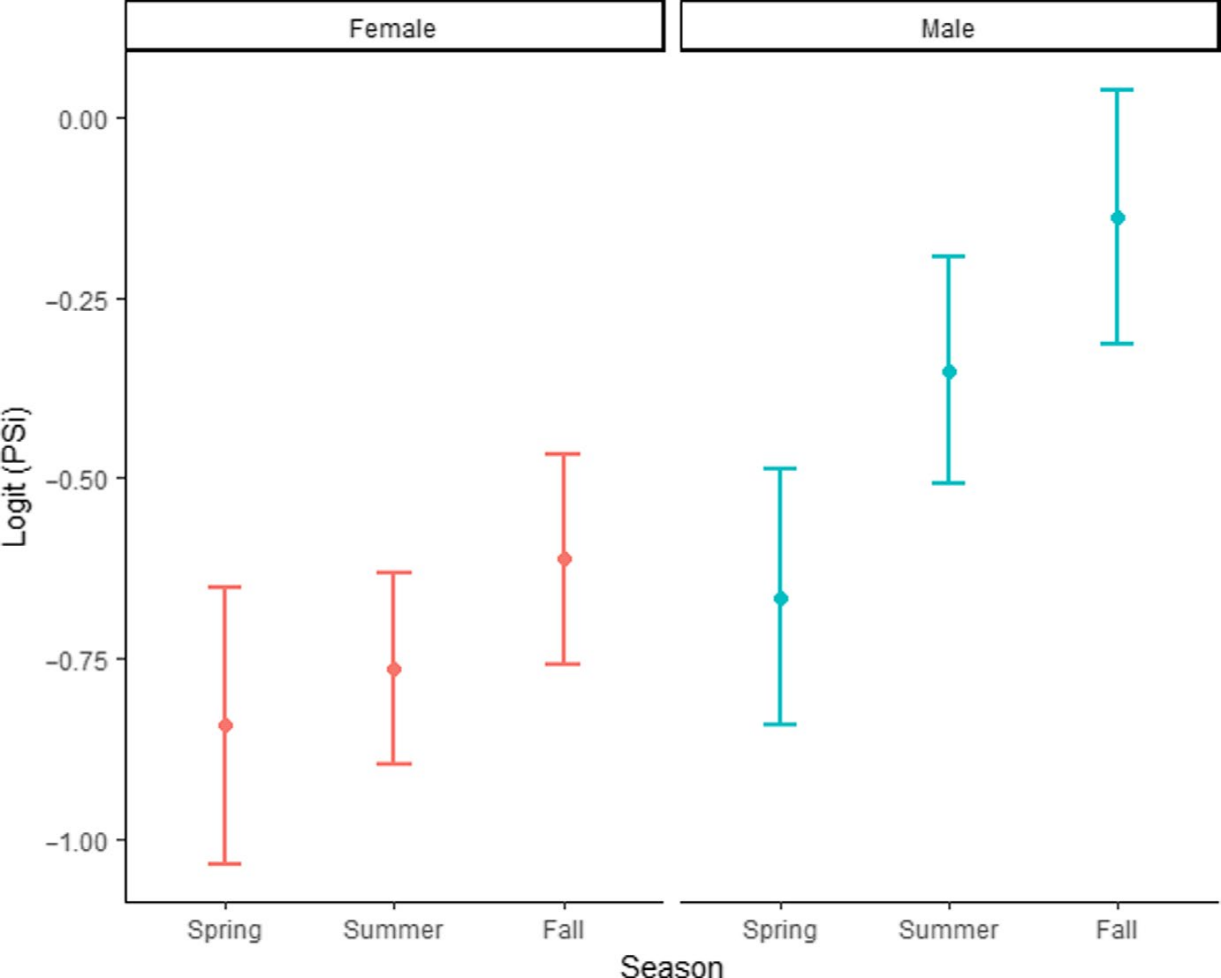
Logit‐transformed average $PS_{i}$ values with 95% confidence intervals of all harbor seal scat from groups with >5 samples (*n* = 1,083 scat samples). Average $PS_{i}$ was calculated for each group. Groups were then split be sex and lumped by season. Spring = April, May; Summer = June, July, August; Fall = September, October, November. A lower value indicates more specialization

**Figure 6:**
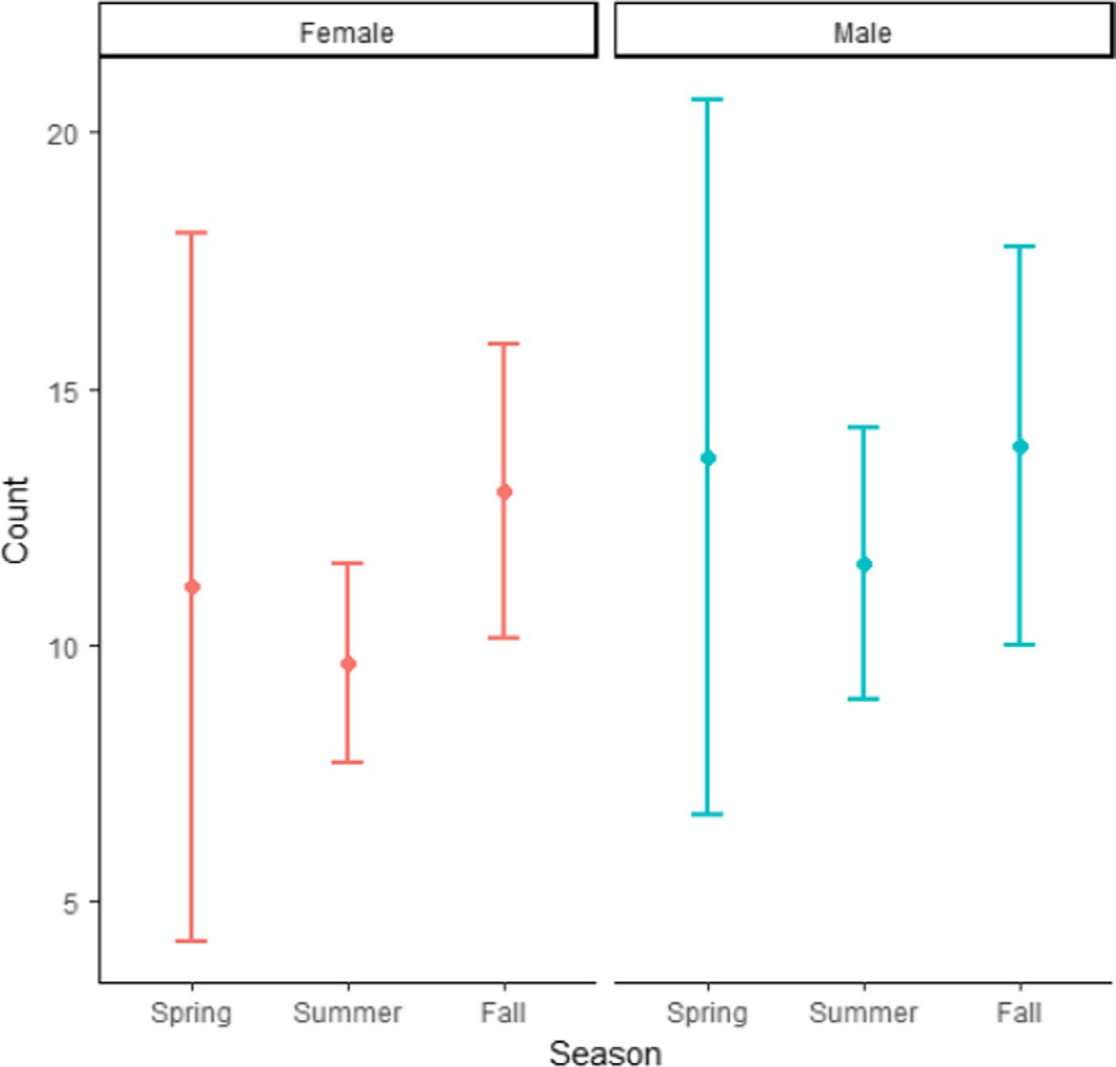
Average sample size with 95% confidence intervals of harbor seal groups for analysis with >5 samples (*n* = 1,083 scat samples). Groups were then split be sex and lumped by season. Spring = April, May; Summer = June, July, August; Fall = September, October, November

To further examine the interaction between Sex and Month, the factor of Month was grouped into three levels: spring (April and May), summer (June, July, August), and fall (September, October, and November). The specialization metric showed a distinct shift throughout the year in males but not females (Figure 5). In summer and fall, males had relatively lower levels of specialization than females (Figure 5). To address the potential bias introduced by sample size for this mode of data analysis, we plotted the sample size for each group by season. The pattern observed in $PS_{i}$ values was not reflected in sample size (Figure 6).

**Figure 7:**
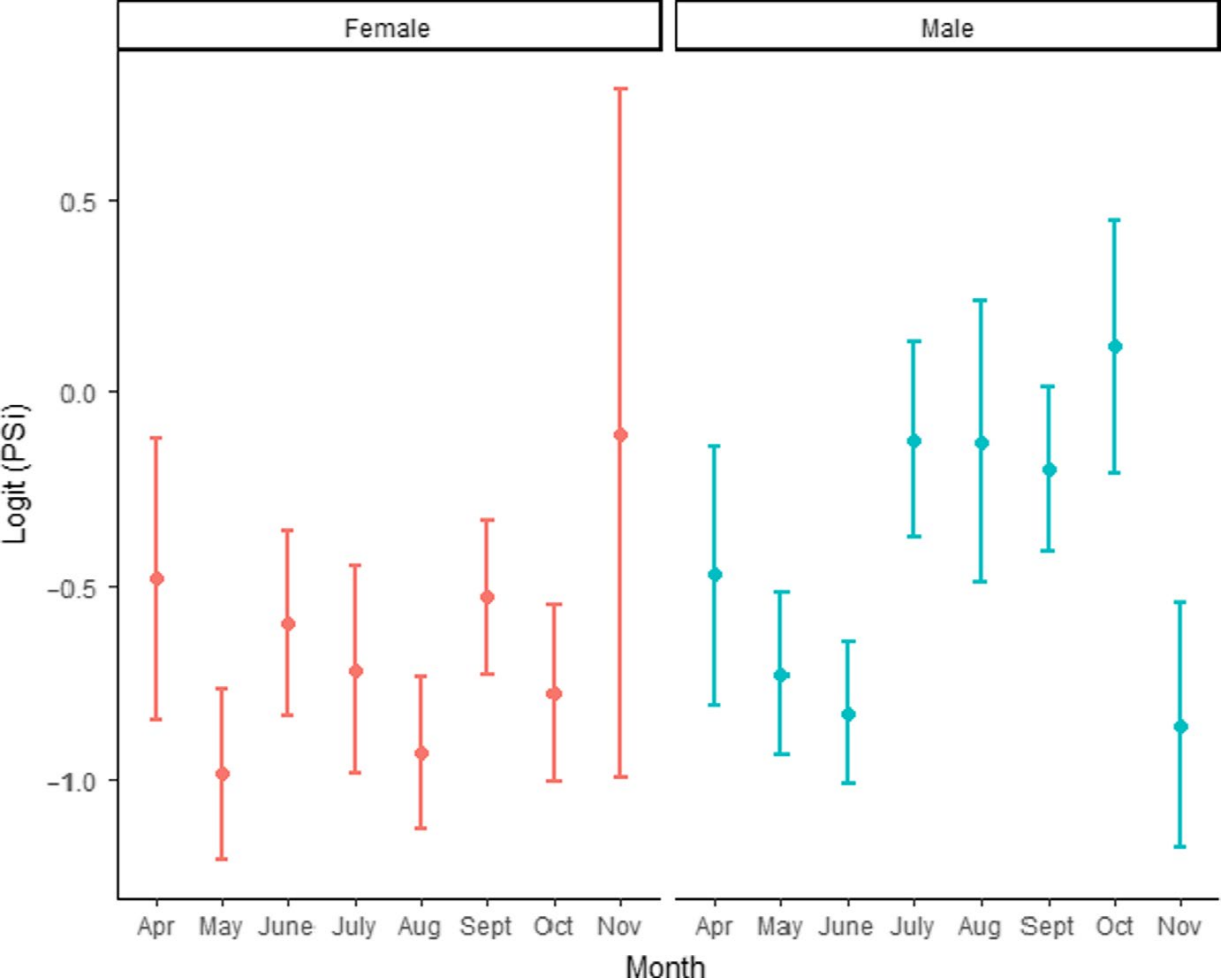
Logit‐transformed average $PS_{i}$ values with 95% confidence intervals of all harbor seal scat from groups with >5 samples (*n* = 1,083 scat samples). Average $PS_{i}$ was calculated for each group. Groups were then split be sex and lumped by Month. The left graph shows females; the right graph shows males. A lower value indicates more specialization

**Figure 8:**
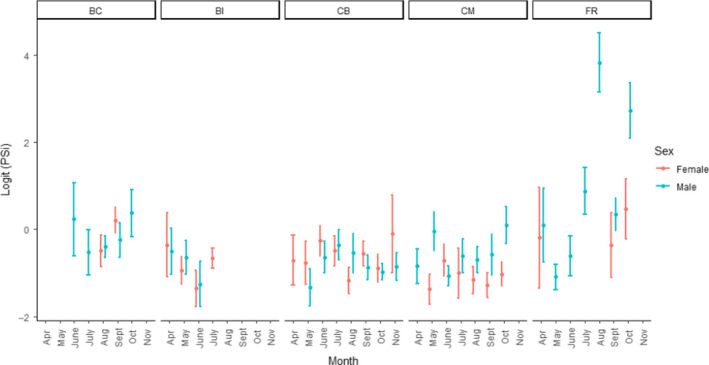
Logit‐transformed average $PS_{i}$values and 95% confidence intervals for all harbor seal scat from groups with >5 samples (*n* = 1,083 scat samples). Average $PS_{i}$was calculated for each group. Groups were then split by sex and location and then lumped by month. A lower value indicates more specialization. BC = Belle Chain, BI = Baby Island, CB = Cowichan Bay, CM = Comox, FR = Fraser River

Visual inspection of the data by month suggested males had a decrease in relative specialization in July through October (Figure 7). Based on 95% confidence intervals of logit‐transformed $PS_{i}$ values, $PS_{i}$ during these months only overlapped with April (Figure 7). The same pattern was not apparent in females because the 95% confidence interval for logit‐transformed $PS_{i}$ of female groups overlapped for all months (Figure 7). This trend varied in intensity by location (Figure 8). The described pattern was reflected most strongly in Belle Chain, Comox, and Fraser River (Figure 8). However, because scat were not collected at Baby Island after July, no comparison could be made at that location (Figure 8, Table 1).

### 3.3 Correlations between prey items and relative specialization

Correlation analysis between diet proportions of prey orders and $PS_{i}$ revealed that ten out of fifteen orders (counting juvenile and adult Salmoniformes as additional separate “orders”) showed significant correlations (alpha = 0.0038, Bonferroni correction, Table 4). Adult Salmoniformes, a largely pelagic group, were correlated with a generalist diet (rho = 0.27, *p* < .001), while juvenile Salmoniformes showed no significant correlation. Salmoniformes (adult and juveniles combined) were also correlated with a generalist diet (rho = 0.17, *p* < .001). Clupeiformes, another largely pelagic group, correlated with a generalist diet as well (rho = 0.24, *p* < .001). Conversely, Pleuronectiformes, a demersal and benthic group, correlated with a specialist diet (rho = −0.38, *p* < .001). Further, Gadiformes, which has both pelagic and demersal representatives, showed no correlation (rho = −0.04, *p* = .38).

**Table 4:** Correlations of prey proportions by taxonomic order to $PS_{i}$ values of Salish Sea harbor seals

Order	rho	*p*	Number of occurrences
Chimaeriformes*	−0.62	<0.001	28
Rajiformes*	−0.52	0.002	32
Scorpaeniformes*	−0.50	<0.001	132
Perciformes*	−0.48	<.001	194
Batrachoidiformes*	−0.40	<.001	82
Pleuronectiformes*	−0.38	<.001	154
Petromyzontiformes	−0.13	.385	44
Osmeriformes	−0.11	.234	128
Gasterosteiformes	−0.07	.53	86
Gadiformes	−0.04	.38	432
Juvenile Salmoniformes	0.03	.523	326
Salmoniformes*	0.17	<.001	612
Adult Salmoniformes*	0.27	<.001	595
Clupeiformes*	0.24	<.001	538
Cephalopoda*	0.39	<.001	163

Note

A Bonferroni correction was used to set alpha at 0.0038. Thus, *p* < .0038 is significant and is denoted by an asterisk next to the order name. A negative rho value indicates a positive correlation with specialization. Analysis represents 1,083 samples from groups with > 5 samples. Data are organized by correlation value.

Correlations performed with just data from female scat showed similar patterns. All orders of prey showed the same relationship with $PS_{i}$, or were no longer significant, such as Adult Salmoniformes, Salmoniformes, Chimaeriformes, Rajiformes, and Batrachoidiformes (Table 5). Correlations performed with only male scat once again showed the same patterns. Orders that no longer had significant relationships were Rajiformes, Batrachoisiformes, Pleuronectiformes, and Clupeiformes (Table 6). However, Adult Salmoniformes and Salmoniformes once again showing significant correlations (Table 6).

**Table 5:** Correlations of prey proportions by taxonomic order to $PS_{i}$ values of Salish Sea female harbor seals

Species	rho	*p*	Number of occurrences
Rajiformes	−0.77	.103	5
Scorpaeniformes*	−0.55	<.001	76
Perciformes*	−0.45	<.001	121
Pleuronectiformes*	−0.44	<.001	70
Batrachoidiformes	−0.41	.013	35
Chimaeriformes	−0.21	.662	6
Osmeriformes	−0.08	.570	51
Gasterosteiformes	−0.07	.662	42
Gadiformes	−0.06	.400	184
Juvenile Salmoniformes	−0.05	.427	359
Petromyzontiformes	0.01	.963	18
Salmoniformes	0.03	.706	529
Clupeiformes*	0.39	<.001	232
Cephalopoda*	0.40	.001	63
Adult Salmoniformes	0.05	.499	339

Note

A Bonferroni correction was used to set alpha at 0.0038. Thus, *p* < .0038 is significant and is denoted by an asterisk next to the order name. A negative rho value indicates a positive correlation with specialization. Analysis represents 498 samples from groups with >5. Data are organized by correlation value.

**Table 6:** Correlations of prey proportions by taxonomic order to $PS_{i}$ values of Salish Sea male harbor seals

Order	rho	*p*	Number of occurrences
Chimaeriformes*	−0.70	<.001	21
Rajiformes	−0.53	.004	26
Perciformes*	−0.52	<.001	72
Batrachoidiformes	−0.41	.004	46
Scorpaeniformes*	−0.40	.002	55
Petromyzontiformes	−0.34	.088	25
Pleuronectiformes	−0.30	.005	83
Osmeriformes	−0.12	.293	76
Gasterosteiformes	−0.05	.726	43
Gadiformes	−0.04	.585	247
Juvenile Salmoniformes	0.001	.985	185
Clupeiformes	0.12	.044	305
Salmoniformes*	0.23	<.001	397
Adult Salmoniformes*	0.33	<.001	316
Cephalopoda*	0.43	<.001	99

Note

A Bonferroni correction was used to set alpha at 0.0038. Thus, *p* < .0038 is significant and is denoted by an asterisk next to the order name. A negative rho value indicates a positive correlation with specialization. Analysis represents 647 samples from groups with > 5 samples. Data are organized by correlation value.

The correlation between proportion of benthic species in each scat and $PS_{i}$ suggested a positive relationship between the amount of benthic species consumed and the level of relative specialization (rho = −0.384, *p* > .001). A similar relationship was observed when the female and male data sets were examined separately (rho = −0.407, *p* > .001; rho = −0.35, *p* > .001, respectively).

## 4 DISCUSSION

We successfully assigned $PS_{i}$ values to 1,083 scat collected from five different locations over the course of four nonsequential years (Table 1, Figure 1). As measured by repeated cross‐sectional sampling and a specialization metric ($PS_{i}$), the overall level of intrapopulation feeding diversity in the region was high ($PS_{i}$ = 0.399, 95% CI = 0.026, *R* = 100,000). Further, Month, Sex, and Location were all important factors influencing this feeding diversity. Interestingly, Month and Sex had a significant interaction. Habitat of an individual's primary prey also seemed to have an impact on relative specialization suggesting that seasonal and sex‐specific patterns in the use of benthic versus pelagic were the underlying cause for the observed intrapopulation feeding diversity. These indications of intrapopulation feeding diversity suggest the feeding ecology of harbor seals in the Salish Sea is complex and that each sex has different impacts on prey species of concern.

### 4.1 Estimated level of intrapopulation feeding diversity

Our data confirmed intrapopulation feeding diversity across the spatial (hundreds of km) and temporal (years) scales that the scat samples represented (average = $PS_{i}$ 0.399, 95% CI = 0.026, *R* = 100,000). These data demonstrate intrapopulation feeding diversity but leave room for two alternative hypotheses (which cannot be separated in this case), regarding the absolute level of individual specialization: the occurrence of short‐term specialists versus long‐term specialists.

### 4.2 *Importance of Month*,* Sex*,* Location*,* and Year on relative specialization* ($PS_{i}$)

Month was an important predictor of relative specialization because removing it from the model caused a large drop in goodness‐of‐fit measurements (Table 3). This pattern makes intuitive sense as the type of prey eaten by harbor seals (Lance et al., 2012; Olesiuk et al., 1990) as well as their dive foraging behavior (Wilson et al., 2014) vary throughout the year. Therefore, changes in foraging behavior (both prey choice and dive type) were likely mechanisms behind the observed change in relative specialization throughout the year. However, there were likely other factors influencing relative specialization in addition to month.

Sex also had an impact on relative specialization, yet smaller than that of Month (Table 2). Differences in the level of relative specialization between female and male harbor seals were likely due to females and males in the region eating different prey items and having different foraging strategies (Bjorkland et al., 2015; Schwarz et al., 2018; Wilson et al., 2014). For instance, females more often perform deeper foraging dives, eat benthic prey more commonly, and have smaller home ranges than males (Peterson et al., 2012; Schwarz et al., 2018; Wilson et al., 2014). Therefore, we propose the following theoretical resource distribution: Males have more overlap between individuals (less specialized), while the females have less overlap between individuals (more specialized) (Figure 5); variations in this pattern appear to be associated with prey type (which will be addressed in Section 4.3).

Including an interaction term between Month and Sex increased the goodness of fit of the model (Table 3). This result indicates that differences between male and female seals likely varied throughout the year. Specifically, there were clear decreases in relative specialization in male harbor seals during the summer and fall months that were not reflected in females (Figure 5), indicating that the behavior of both sexes was similar in the spring but diverged in the summer and fall. During the late summer and fall, there is a large influx of returning adult Salmoniformes (Quinn, 2005) that are preyed upon by both female and male harbor seals (Schwarz et al., 2018). In the Salish Sea, Salmoniformes can compose >50% of the population diet in the summer and fall (Lance et al., 2012). This resource could be rich enough that it is beneficial for a majority of seals, resulting in less need for specialization. Further, males consume more Salmoniformes than females (Shwarz et al., 2018) which could result in the larger spike observed in generalist behavior in males.

Our data also suggest that location explained a large amount of variance in relative specialization. The random factors of Year and Location increased the $r^{2}$ by more than four times, indicating that both had a large influence on relative specialization. However, because Sample Size, Location, and Year explained 0.39, 0.36, and 0.002 of the variance (*SD* = 0.62, 0.597, 0.05), respectively, one can assume that Sample Size and Location were the random factors responsible for the increase in goodness of fit of the model, not Year. This result indicates that where the seals were foraging impacted the level of relative specialization in the population, without noticeable changes from year to year. Our results also indicate that there was likely some bias introduced by the number of samples in a group. For instance, there was a correlation between average $PS_{i}$ and theoretical minimum $PS_{i}$ (rho = −0.231, *p* = .03). However, this potential bias is unlikely to have had a substantial effect on the outcome of our study because we included sample size as a random variable in the model and variation in sample size does not appear to explain the seasonal changes in $PS_{i}$ (Figures 5, 6).

The importance of location as a factor in explaining variation of relative specialization could be due to varied levels of prey diversity in different environments, given that prey availability affects the level of specialization (Araújo et al., 2011). For example, harbor seal scats from haul‐outs in estuaries have higher prey diversity than scat from haul‐outs outside estuaries (Lance et al., 2012; Luxa & Acevedo‐Gutiérrez, 2013). Cowichan and Fraser River are situated within estuaries, Baby Island and Comox are located near both estuarine and nonestuarine habitats, and Belle Chain is considered a rocky reef environment. Therefore, it is likely that variation in habitat types caused differences in prey availability that offered more or less options to harbor seals in the area, which could subsequently affect the level of competition and, ultimately, specialization. The spatiotemporal variation in relative specialization throughout the region suggests widespread intrapopulation feeding diversity. This knowledge can inform the design of future studies and act as a starting place to investigate the impacts of intrapopulation feeding diversity on prey species in the ecosystem.

### 4.3 Correlation between relative specialization and prey species composition

Our data suggest that the higher proportion of benthic species consumed, the relatively more specialized the diet of the predator became. This pattern was observed in the full dataset, as well as when female and male data were considered separately (Tables 4, 5, 6). This information ties to our knowledge of the foraging patterns of male versus female harbor seals in the region. Females more often perform deeper foraging dives (Wilson et al., 2014) and eat more benthic species than males, who eat more pelagic species (Schwarz et al., 2018). In Scotland, harbor seal scat samples represented either a largely pelagic foraging strategy or largely benthic foraging strategy (Tollit, Greenstreet, & Thompson, 1997), and males had larger range and duration in foraging trips (Thompson, Mackay, Tollit, Enderby, & Hammond, 1998), suggesting that the separation between the two foraging strategies is not just a regional phenomenon.

The described pattern of benthic prey correlation with relative specialization seems to hold true for both females and males (Tables 4, 5, 6). This result indicates that specialization patterns linked to prey species were reflective of foraging strategies specific to the ecology of prey species, and not just indicative of different diet preferences of males and females. We hypothesize that this pattern was caused by higher variability in benthic environments (Lalli & Parsons, 1997). If prey have more variable life strategies, a single foraging strategy will not suffice to catch them all. Because an organism is likely limited in the number of foraging strategies at which it can be effective, an individual could be limited in the number of prey species it can exploit.

There is the possibility that the sex of the individual determines the level of specialization regardless of the prey consumed. However, we argue that sex determines the feeding strategy to be employed and that the prey species captured by each feeding strategy determines the level of specialization. The consistency of benthic prey being associated with a relatively specialist diet, and pelagic prey being associated with a relatively generalist diet in our three datasets (complete, only female, and only male) suggests that the ecology of the prey species rather than the sex of the seal was driving the observed pattern. This idea is supported by other studies. Individual male harbor seals in Nova Scotia use different behaviors when foraging for benthic versus pelagic prey (Bowen, Tully, Boness, Bulheier, & Marshall, 2002). Further, large harbor seals are more likely to forage in pelagic environments regardless of sex (Bjorkland et al., 2015). Because harbor seals display slight sexual dimorphism, with females being slightly smaller than males (Teilmann & Galatius, 2018), there is the potential for trade‐offs between speed and maneuverability within the population. If that is the case, then females would be slightly slower and more maneuverable than males. We hypothesize that this combination of traits would be more successful in a benthic environment to deal with variations in the seafloor that benthic prey relies on to escape. If this idea is correct, then the sex of an average harbor seal predisposes an individual to be more effective in one type of environment than the other.

If prey species ecology is driving specialization levels, it is especially interesting to consider harbor seal consumption of juvenile Salmoniformes. As a group, juvenile Salmoniformes did not correlate with the relatively more generalist diet. However, when split into species, juvenile sockeye (*Oncorhynchus nerka*) did correlate with this diet (rho = 0.22, *p* = .004). This correlation could indicate that seals were not seeking out juvenile Salmoniformes specifically but rather eating them as a byproduct of focusing on fish that match the image of forage fish (e.g., small and silver) while conducting a pelagic foraging strategy. This is just one example of how understanding the level of specialization could deepen our scope of knowledge regarding harbor seal impacts on prey species of concern.

### 4.4 Study limitations

There are a few notable limitations to this study. First, there was the potential for variation in sample size to introduce bias. However, there were no discernible patterns between sample size and average relative specialization by season (Figures 2, 3). We also included sample size as a random factor in the model to account for any bias introduced there. Hence, any bias introduced by sample size was likely minimal. Second, because scat were collected from the same haul‐out multiple times, it is possible that some scat collected came from the same individual. However, this possibility is small given than Rothstein et al. (2017) estimated the sampling scheme to track five individuals at Cowichan Bay (i.e., a single haul‐out) as 440 samples over 22 sampling bouts. Compared to the 1,083 samples used in this analysis from five different haul‐outs, it seems unlikely there was a high rate of resampling the same individuals. Third, there are biases in the metabarcoding PCR process for determining diet (Thomas, Jarman, Haman, Trites, & Deagle, 2014). The prey proportions recorded for each sample are not directly proportional to the amount of prey that was ingested (Bowen & Iverson, 2012; Thomas et al., 2014). However, this approach is accepted to be semi‐quantitative, biases appeared to be consistent between samples (Thomas et al., 2014), and the approach has been successfully used in other studies (Deagle et al., 2010; Pompanon et al., 2012; Schwarz et al., 2018; Thomas et al., 2014, 2017). Furthermore, this approach is superior to the alternative occurrence‐based methods for generating diet proportions (Deagle et al., 2019). On a related note, these molecular methods do not provide data that directly equate to counts of prey consumed. But, if individuals within a local group encounter the same size distribution of a given prey species, then diet proportions represent the same relative relationship of prey capture decisions. Further investigation into potential biases introduced by using proportion type data would be useful as this methodology has many benefits and is a valuable molecular technology that should be applied in the future.

## 5 CONCLUSION

We have shown that intrapopulation feeding diversity occurs in Salish Sea harbor seals between locations, seasons, and sexes. Females displayed higher levels of relative specialization than males. However, in both female and male harbor seals, benthic prey were more commonly associated with a more specialized diet, suggesting the prey's ecology has an important role in driving the level of specialization. These different impacts of male versus female on benthic versus pelagic prey should be considered henceforth when management addresses harbor seal interactions with species of concern. Further, we demonstrated how the use of molecular prey barcoding from scat allows for high taxonomic and spatiotemporal resolution of relative individual specialization. The resulting large‐scale examinations of intrapopulation feeding diversity uncovered previously unknown complex interactions between predators and prey.

## CONFLICT OF INTEREST

The authors declare no conflict of interest.

## AUTHOR CONTRIBUTION

**Madelyn Voelker:** Conceptualization (equal); Data curation (lead); Formal analysis (lead); Funding acquisition (supporting); Investigation (lead); Methodology (equal); Project administration (equal); Resources (lead); Writing‐original draft (lead); Writing‐review & editing (lead). **Dietmar Schwarz:** Conceptualization (equal); Data curation (supporting); Formal analysis (supporting); Funding acquisition (supporting); Investigation (supporting); Methodology (equal); Project administration (supporting); Resources (supporting); Supervision (equal); Writing‐original draft (supporting); Writing‐review & editing (equal). **Austen Thomas:** Conceptualization (supporting); Data curation (supporting); Formal analysis (supporting); Methodology (supporting); Resources (supporting); Writing‐review & editing (supporting). **Benjamin Nelson:** Data curation (supporting); Formal analysis (supporting); Investigation (supporting); Methodology (supporting); Resources (supporting); Writing‐review & editing (equal). **Alejandro Acevedo‐Gutiérrez:** Conceptualization (equal); Data curation (supporting); Formal analysis (supporting); Funding acquisition (lead); Investigation (equal); Methodology (equal); Project administration (equal); Resources (supporting); Supervision (equal); Writing‐original draft (supporting); Writing‐review & editing (equal).

## Data Availability

All diet data used are publicly available. The sequence data are stored at: https://figshare.com/s/0113457d4081727aacac (Thomas et al., in review). The breakdown of prey proportion within each scat as well as sex assignment for these samples is located on Dryad (10.5061/dryad.59zw3r257).
